# The MedConnect Program: Symptomatology, Return Visits, and Hospitalization of COVID-19 Outpatients Following Discharge From the Emergency Department

**DOI:** 10.7759/cureus.26771

**Published:** 2022-07-12

**Authors:** Bryana L Bayly, Jacquelyn B Kercheval, James A Cranford, Taania Girgla, Arjun R Adapa, Ginette V Busschots, Katheen Y Li, Marcia Perry, Christopher M Fung, Colin F Greineder, Eve D Losman

**Affiliations:** 1 Psychiatry, Brigham and Women’s Hospital, Boston, USA; 2 Internal Medicine, Duke University, Durham, USA; 3 Emergency Medicine, University of Michigan Health/Michigan Medicine, Ann Arbor, USA; 4 Emergency Medicine, University of Michigan Medical School, Ann Arbor, USA; 5 Emergency Medicine, Icahn School of Medicine at Mount Sinai, New York, USA

**Keywords:** medical student, emergency medicine and trauma, followup, covid 19, discharge to home

## Abstract

Background and objective

Although hospitalization is required for only a minority of those infected with severe acute respiratory syndrome coronavirus 2 (SARS-CoV-2), the high rates of morbidity and mortality among these patients have led researchers to focus on the predictors of admission and adverse outcomes in the inpatient population. However, there is scarce data on the clinical trajectory of individuals symptomatic enough to present for emergency care, but not sick enough to be admitted. In light of this, we aimed to examine the symptomatology, emergency department (ED) revisits, and hospitalization of coronavirus disease 2019 (COVID-19) outpatients after discharge from the ED.

Methods

Adult patients with COVID-19 infection were prospectively enrolled after discharge from the ED between May and December 2020. Patients were followed up longitudinally for 14 days via phone interviews designed to provide support and information and to track symptomatology, ED revisits, and hospitalization.

Results

A volunteer, medical student-run program enrolled 199 COVID-19 patients discharged from the ED during the first nine months of the pandemic. Of the 176 patients (88.4%) who completed the 14-day protocol, 29 (16.5%) had a second ED visit and 17 (9.6%) were admitted, 16 (9%) for worsening COVID-19 symptoms. Age, male sex, comorbid illnesses, and self-reported dyspnea, diarrhea, chills, and fever were associated with hospital admission for patients with a subsequent ED visit. For those who did not require admission, symptoms generally improved following ED discharge. Age >65 years and a history of cardiovascular disease (CVD) were associated with a longer duration of cough, but generally, patient characteristics and comorbidities did not significantly affect the overall number or duration of symptoms.

Conclusions

Nearly one in five patients discharged from the ED with COVID-19 infection had a second ED evaluation during a 14-day follow-up period, despite regular phone interactions aimed at providing support and information. More than half of them required admission for worsening COVID-19 symptoms. Established risk factors for severe disease and self-reported persistence of certain symptoms were associated with hospital admission, while those who did not require hospitalization had a steady improvement in symptoms over the 14-day period.

## Introduction

Since its emergence in late 2019, the coronavirus disease 2019 (COVID-19) pandemic has spread to nearly every region of the world and has had a devastating impact on individual health and healthcare delivery. In the US, high volumes of unscheduled visits and limited hospital and ICU capacity put such an unprecedented burden on emergency care providers in 2020 that they struggled to accurately discern which patients required admission and/or critical care [1-3]. Consequently, most early research efforts focused on identifying the severe disease and predicting adverse outcomes in those hospitalized with COVID-19 [4-7].

However, there is very little data on individuals with relatively mild disease. Of particular interest is the subset of patients whose symptoms are severe enough to prompt a visit to the emergency department (ED), but whose clinical condition is ultimately deemed stable enough to allow a discharge home. A retrospective study during the first three months of the pandemic found that approximately 9% of these patients had another unscheduled ED visit within 72 hours [8]. Similarly, a multicenter study aimed at developing a predictive model for identifying those at risk of a return visit and hospitalization found that 21% of patients had returned to the ED by 30 days from the index ED visit [9]. Unfortunately, the design of these studies precluded the determination of what percentage of repeat visits were related to COVID-19 or what factors may have been responsible for the high rate of return (e.g., worsening symptoms, inadequate follow-up, and anxiety). Nonetheless, efforts have been made to monitor patients remotely in the hope of identifying those with evidence of disease progression, and therapeutic strategies have targeted “high-risk” patients discharged from the ED in an effort to reduce subsequent hospitalization [10-12]. A better understanding of symptom progression in these patients and the impact of various demographics and/or comorbidities might improve these efforts and assist providers caring for these patients following an ED visit.

To address this knowledge gap, we describe the MedConnect program, a medical student-driven effort initiated in the early phase of the pandemic aimed at maintaining close contact with patients after discharge from the ED. Leveraging data gathered via phone interviews, we report on the clinical trajectory of nearly 200 COVID-19 patients discharged from the ED of a single institution in the US.

## Materials and methods

Human subjects approval

This work was reviewed and deemed exempt by the University of Michigan Institutional Review Board; HUM00182252.

The MedConnect program: patient enrollment and overview

MedConnect was developed by two fourth-year medical students at the University of Michigan Medical School (BLB and JBK) in partnership with a faculty advisor (EDL) and was launched in May 2020. The purpose of the program was to provide frequent symptom monitoring, support, and information to COVID-19 patients following their discharge from the University of Michigan Adult ED.

In our ED, criteria for the discharge of patients with suspected or known COVID-19 infection were established by the expert consensus of our hospital's Infectious Disease specialists. If a patient was not at high risk for complications (age >70 years, pregnant, immunocompromised) and had mild respiratory symptoms, fever controlled with antipyretics, and normal vital signs (heart rate <100, room air oxygen saturation >92% at rest and with ambulation), they were discharged home. In our hospital, in the early phase of the pandemic, we had no mechanism to obtain pulse oximeters for home use. We relied on patient reports of their symptoms and symptom severity.

Every 24 hours, an automated list was generated using an electronic health record (EHR) query of patients aged 18 years or older who were discharged from the ED with a positive COVID-19 nasopharyngeal swab either during that ED visit or in the preceding 14 days. The automated report identified patients with previously positive tests, including those performed in the outpatient setting or at other hospitals, because of a hospital policy during the study period which mandated that all symptomatic individuals arriving at the ED had to either submit to real-time PCR testing or provide documentation of a positive result with an equivalent PCR-based test. All flagged charts were reviewed by one of eight trained medical students to confirm each patient’s age, diagnosis of COVID-19 within the preceding 14 days, and discharge from the ED. Eligible patients were then called, this history was confirmed, and patients were offered enrollment in the MedConnect program. The patients were read a standard research informed consent form and, if they agreed, it was emailed to them; the patients provided verbal consent to participate. There was no incentive offered to join the program. If the patient did not answer the initial phone call, a voicemail was left offering the patient the opportunity to return the call. If the patient did not return this call, students made one additional phone call 24 hours later.

Once enrolled, each patient was randomly assigned to one of the eight medical students, who contacted the patient by phone every other day for a 14-day period. This period of observation was selected based on the limited data available at the time suggesting that recovery from acute infection for outpatients typically occurred in 10-14 days. To track and record symptoms in a standardized manner, students were provided with a call script [13]. Patients were asked about the same list of symptoms during each call (fever, cough, dyspnea, body aches, chills, headache, sore throat, and loss/change in taste and/or smell) and data was recorded in real-time. Return visits to the University of Michigan ED or any other local ED were recorded. If the patient was hospitalized, this was documented and their participation in the MedConnect program was terminated. Patients who returned to the ED but were again discharged home remained enrolled in the program. If the patients did not answer any given call, students left voicemails and attempted to call every 24 hours. Patients who did not answer the phone for three consecutive days were considered lost to follow-up and excluded from the analysis.

In addition to symptom tracking, the MedConnect program provided patients with support and care. Students answered patients’ questions, which often revolved around management of symptoms, quarantining/isolating and social distancing, and, where appropriate, returning to work. Each student was partnered with an emergency medicine (EM) faculty member with whom the student would discuss any concerning symptoms or unanswered questions raised by the patient. If appropriate, students contacted the patients again to discuss the recommendations of the EM faculty physician. A note template was used to document this care in the electronic medical record.

Manual chart abstraction

While symptomatology, demographic, and clinical data (including age, gender, race, BMI, and comorbidities) were collected during patient phone calls, we addressed missing data and potential recall bias by validating phone interview data against the information in the EHR. Beyond our primary hospital’s EHR, we leveraged the Great Lakes Health Connect portal to link to the EHRs of 117 area hospitals. Abstraction included a review of patient comorbidities and, for all return visits to the ED, confirmation that the reason for return was the progression of COVID-19 symptoms. Chart abstractions were performed by two EM faculty members with discrepancies adjudicated by a third review. To confirm the accuracy of abstracted data related to ED revisit and admission, a secondary review by one of the EM faculty members (EDL or CFG) was performed.

Data analysis and statistics

Data from each patient’s symptom log were independently coded for analysis by two students. Any differences were compared and reconciled by a third student. Continuous dependent variables were analyzed using independent-groups t-tests and one-way analyses of variance (ANOVAs), and categorical dependent variables were analyzed using chi-squared tests. Post-hoc comparisons using Tukey HSD (honestly significant difference) were also performed. An alpha level of 0.05 was used for all analyses.

## Results

During the study period from May 4, 2020, to December 31, 2020, 347 patients met the criteria based on chart review; 199 (52.9%) enrolled, 69 (19.9%) declined enrollment, and 78 (22.5%) were unable to be reached. Of the 199 enrolled, 176 (88.4%) completed the full protocol, while 23 (11.6%) were lost to follow-up. There were no statistically significant differences between these groups in terms of age (enrolled: 45.0 ± 15.9 years; not reached: 45.1 ± 16.8 years; declined: 43.9 ± 17.3 years; and enrolled but excluded: 37.1 ± 11.5 years), race (Asian, Black or African American, White or Caucasian, Other), or ethnicity (Hispanic or non-Hispanic). There was a higher percentage of females in the enrolled (60.8%) and not reached (64.1%) groups compared to the declined (42.0%) and enrolled but excluded (47.8%) groups.

Symptom description and predictors of admission among patients who were eventually admitted following initial discharge from the emergency department

Of the 176 patients who enrolled in the study and completed the follow-up protocol, 29 (16.5%) returned to an ED within 14 days of their initial ED visit. Seventeen (9.6%) of those patients were admitted, with an average duration of 5.7 ± 2.8 days from the initial ED visit until admission. Most (11/17, 64.8%) patients were admitted within the same hospital system, while 6/17 (41.2%) went to an outside hospital. All but one (94.1%) were admitted for worsening COVID-19 symptoms, while the remaining patient was hospitalized for acute coronary syndrome and found to have an occluded bypass graft. Two additional patients were found to have returned for admission shortly after the end of the 14-day MedConnect program (16 and 18 days after their initial ED visit, respectively). Neither of those hospitalizations was related to COVID-19 (one for gastrointestinal hemorrhage and the other for *Streptococcus mitis* bacteremia).

To better understand the course of COVID-19 infection in patients discharged from the ED, we focused our analyses on three cohorts of patients: those who (a) subsequently returned to the ED and were admitted (n=17), (b) returned to the ED but did not require hospitalization (n=12), and (c) did not return to the ED in the 14-day period (n=147). We first assessed whether there was any difference in reporting of the eight standardized symptoms between each cohort. Those who were admitted reported more total symptoms on average (6.2 ± 1.9) than the other groups (returned but not admitted: 4.8 ± 2.2; did not return: 4.6 ± 2.3, p=0.04). The difference between the latter groups was not statistically significant (p=0.70).

Next, we compared individual symptoms and the percentage of patients in each cohort who reported them during follow-up phone calls. As shown in Table 1, an ANOVA with patient cohort (returned to ED and admitted, returned to ED but not admitted, and no return to ED) as the between-groups factor showed a statistically significant effect on the prevalence of multiple symptoms: dyspnea (88.2% vs. 75.0% vs. 53.7%, p=0.01), diarrhea (77.6% vs. 16.7% vs. 48.3%, p=0.02), chills (76.5% vs. 58.3% vs. 43.5%, p=0.03), and fever (52.9% vs. 41.7% vs. 26.5%, p=0.05).

**Table 1:** Prevalence of symptoms in the three groups Symptoms are ordered as per the prevalence of appearance in the returned to ED, admitted group ED: emergency department; SD: standard deviation

Symptoms	Returned to ED and admitted (n=17)	Returned to ED, not admitted (n=12)	No return to ED (n=147)	P-value
Cough	16 (94.1%)	9 (75.0%)	116 (78.9%)	0.30
Dyspnea	15 (88.2%)	9 (75.0%)	79 (53.7%)	0.01
Diarrhea	12 (77.6%)	2 (16.7%)	71 (48.3%)	0.02
Chills	13 (76.5%)	7 (58.3%)	64 (43.5%)	0.03
Headache	13 (76.5%)	6 (50.0%)	94 (63.9%)	0.34
Body aches	11 (64.7%)	8 (66.7%)	98 (66.7%)	0.99
Dys-/anosmia or dys-/ageusia	11 (64.7%)	5 (41.7%)	94 (63.9%)	0.30
Fever	9 (52.9%)	5 (41.7%)	39 (26.5%)	0.05
Sore throat	6 (35.3%)	4 (33.3%)	56 (38.1%)	0.93
Total number of symptoms, mean (SD)	6.2 (1.9)	4.6 (2.3)	4.8 (2.2)	0.04

Interestingly, these four symptoms - dyspnea, diarrhea, fevers, and chills - were the same ones consistently noted by our medical student volunteers as causing the patients the most distress and about which patients most frequently asked for advice during phone interviews. Post-hoc comparisons using Tukey HSD revealed significant differences between the returned to ED and admitted cohorts and the cohort that did not return to the ED. None of the other between-group comparisons were statistically significant.

Patient characteristics and comorbidities were then compared between the three cohorts (Table 2).

**Table 2:** Demographic and clinical characteristics of the three groups ED: emergency department; SD: standard deviation; BMI: body mass index

Variables	Returned to ED and admitted (n=17)	Returned to ED, not admitted (n=12)	No return to ED, not admitted (n=147)	P-value
Age, years, mean (SD)	54.3 (16.3)	48.9 (15.8)	43.8 (15.6)	0.02
Male gender, n (%)	12 (70.6%)	5 (41.7%)	53 (36.1%)	0.02
Race, n (%)				0.78
White	12 (70.6%)	10 (83.3%)	105 (71.4%)	
Black	2 (11.8%)	1 (8.3%)	27 (18.4%)	
Other	3 (17.6%)	1 (8.3%)	15 (10.2%)	
BMI, kg/m^2^, mean (SD)	32.2 (7.3)	29.0 (6.5)	32.8 (8.2)	0.35
Charlson Comorbidity Index total score, mean (SD)	3.0 (3.3)	1.9 (2.1)	1.1 (2.0)	0.001
Cardiovascular disease, n (%)	8 (47.1%)	3 (25.0%)	20 (13.6%)	0.002
Chronic obstructive pulmonary disease, n (%)	9 (52.9%)	4 (33.3%)	40 (27.2%)	0.09
Diabetes, n (%)	4 (23.5%)	2 (16.7%)	17 (11.6%)	0.36

As with the aforementioned symptom analysis, stratification by patient cohort also showed a statistically significant association between cohorts and multiple patient characteristics, including age (54.3 vs. 48.9 vs. 43.8 years, p=0.02), sex (70.6% vs. 41.7% vs. 36.1% male, p=0.02), Charlson Comorbidity Index (3.0 vs. 1.9 vs. 1.1, p=0.001), and history of cardiovascular disease (CVD) (47.1% vs. 25.0% vs. 13.6%, p=0.002). Again, post-hoc comparisons showed statistically significant differences between the returned to ED and admitted group vs. the no return to ED group for each of these demographic and clinical characteristics, but there were no statistically significant differences between (a) the returned to ED and admitted group vs. the returned to ED but not admitted group, and (b) the no return to ED group vs. the returned to ED but not admitted group.

Symptom description and predictors of symptom resolution among patients who were not admitted for COVID-19 during the course of the MedConnect program

**Figure 1:**
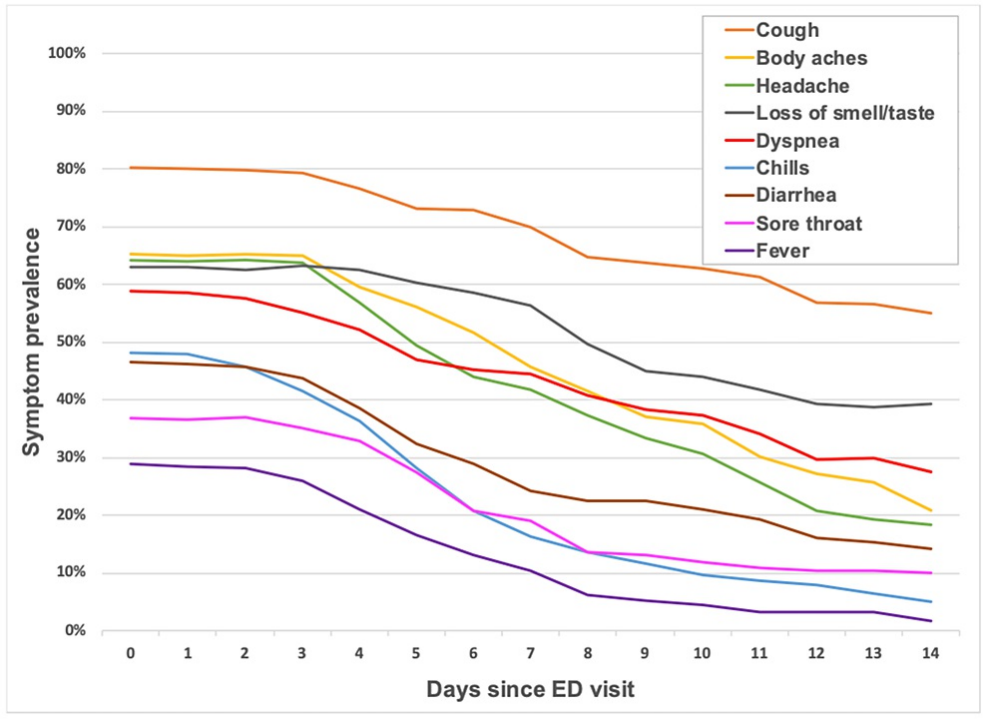
Daily symptom prevalence since index visit to ED in patients not requiring hospital admission (n=159) ED: emergency department

In addition to the cohort analysis, we also examined the pattern of symptom resolution in the 159 patients who did not require admission. Figure 1 shows the prevalence of each symptom over the 14-day period of the MedConnect program.

Overall, patients reported a steady and fairly rapid decline in their symptoms, with only cough present in >50% of patients by the end of the follow-up period. Notably, three of the four “most distressing” symptoms (based on phone interviews by medical students) - diarrhea, fevers, and chills - declined to less than 25% prevalence by one week after the initial ED visit.

Given the associations found between patient demographics and comorbidities and eventual hospital admission, we then tested for similar associations with the number and duration of symptoms in patients who did not require hospitalization. As shown in Table 3, there were no statistically significant effects of age, sex, or history of CVD or chronic obstructive pulmonary disease (COPD) on the overall number of symptoms or the duration of four “most distressing” symptoms: dyspnea, diarrhea, fever, and chills. Indeed, the only statistically significant effects were seen with cough, the most prevalent symptom. Age ≥65 years was statistically significantly associated with longer cough duration (15.2 ± 2.6 vs. 12.5 ± 4.8, p=0.02). Likewise, the duration of cough was statistically significantly longer among those with CVD (15.3 ± 3.4 vs. 12.6 ± 4.7 days, p=0.04).

**Table 3:** Mean number and duration of symptoms in patients not requiring hospital admission (n=159) following discharge from the ED ED: emergency department; CVD: cardiovascular disease; COPD: chronic obstructive pulmonary disease

Variables	Age			Sex			CVD			COPD	
	18-64 years	65 years and older		Female	Male		No	Yes		No	Yes
No. of symptoms	5.0	4.8		5.1	4.7		4.9	5.2		4.9	5.2
Duration of symptoms, days											
Cough	12.5	15.2		12.9	12.9		12.6	15.3		13.1	12.1
Dyspnea	11.3	12.9		11.2	11.6		11.4	11.8		11.8	10.7
Diarrhea	9.4	8.3		9.9	9.0		9.0	10.9		8.9	10.2
Fever	7.1	5.7		6.7	7.3		6.8	7.8		6.9	7.0
Chills	7.5	6.3		7.8	6.4		7.1	7.8		6.9	8.3

## Discussion

From the earliest reports of COVID-19, it has been clear that the illness has a heterogeneous severity. Certain patients, typically those with advanced age and pre-existing respiratory or cardiovascular conditions, are at substantially higher risk of severe disease and adverse outcomes. The findings from the MedConnect program add to the growing body of literature suggesting that these patient factors are also associated with the progression of disease in patients who initially present to the ED with relatively mild symptoms. In our cohort, older age, male sex, greater comorbid illnesses (as judged by a higher Charlson Comorbidity Index), and history of cardiovascular disease were associated with a higher likelihood of admission on a second ED visit. Not surprisingly, these patients also had a greater burden of symptoms and a higher likelihood of dyspnea, diarrhea, fever, and chills. It is worth noting that our study tracked these symptoms prospectively in the days following the patients’ initial ED visit, rather than at the time of initial ED presentation. Whether or not patient symptoms in the ED predict subsequent return or admission was not addressed by MedConnect.

Our findings also support previous research suggesting a high rate of return of COVID-19 patients to the ED. The 16.5% return visit and 9.6% admission rate in our cohort are in line with previous reports from single-hospital systems [14]. Kilaru et al. (2020) reported lower return (8.6%) and admission (4.7%) rates, but their study only assessed patients up to 72 hours and, unlike our approach, could not capture patients who went outside their hospital system. Similarly, Haag et al. (2021) reported only a 9% return rate up to 30 days, although this study enrolled patients not only from EDs but also assessed a mix of urgent care and drive-through-testing facilities. Beiser et al. (2021) recently published a multicenter, ED-based study involving 116 hospitals and found a 21% return visit rate at 30 days, with 7.6% of patients admitted, although these figures were not restricted to COVID-19-related repeat visits or admissions. Despite the similarity in quantitative results, our phone interview-based study suggests multiple potential confounders in retrospective approaches to these questions, especially when the time window from the initial ED visit is extended beyond two weeks. Firstly, only 64% of the 17 patients who were ultimately hospitalized returned to a facility within our health system, suggesting that EHR-based queries may miss a significant proportion of repeat visits. Second, we identified two patients in our cohort who were admitted just outside our 14-day follow-up timeframe, as they were still in phone contact with volunteer medical students. In both cases, chart review revealed that the patients were hospitalized for unrelated illnesses. While not necessarily representing a complete sampling of this group, the results are notable given the contrast with those admitted within 14 days of the index ED visit, ~95% of whom were hospitalized for worsening COVID-19 symptoms.

In addition to the rates and characteristics of patients who returned for admission, the other main finding of the MedConnect study is the steady and relatively rapid improvement in symptoms in those who recovered without needing hospitalization (Figure 1). By one week after the initial ED visit, the prevalence of all the symptoms tracked in our phone interviews had fallen to less than 50%, with the exception of cough and loss/change of taste and/or smell. Our results are somewhat similar to those of Blair et al. (2021), who conducted a phone interview-based study among COVID-19 outpatients, although a direct comparison of the two studies is challenging since the Blair study was indexed to the time of symptom onset, rather than the date of an index ED visit [15]. While the Blair study did not separately examine symptomatology in patients who recovered without the need for hospitalization, the overall pattern of symptom resolution was similar, albeit with consistently lower symptom prevalence in their cohort. Cough, for example, was present in only 58.8% of their patients, as compared to 79.0% in our study, suggesting that patients post-ED discharge may have a distinct symptom trajectory compared to unselected outpatients with positive severe acute respiratory syndrome coronavirus 2 (SARS-CoV-2) PCR testing.

Limitations

Our study has a number of limitations. Although many of our findings reached statistical significance, their interpretation is limited by the small sample size (n=176), and our study may not have detected significant differences in some variables. Likewise, MedConnect was conducted at a single academic hospital system, and our findings may not be generalizable to other geographic locations, practice settings, or patient populations. The study was also temporally limited to the first nine months of the pandemic and may have limited applicability to subsequent waves or virus variants. Participation in the study was voluntary and relied on the ability of medical student volunteers to contact patients by phone. Approximately 42% of eligible patients either declined enrollment or were unable to be reached, and another 6.6% were excluded due to the failure to complete the 14-day phone interview protocol, introducing a risk of selection bias in the final cohort. Likewise, there is always a risk of recall bias in a study based on phone interviews conducted on the days following an ER visit, although we addressed this to some extent by validating phone interview data against the information in the EHR. Finally, since the MedConnect program was a patient care initiative as well as a research effort, it is possible that involvement in the program altered symptomatology or patient decision-making. Indeed, one of the stated goals of the program was to reduce the need for return ED visits by providing patients with support, comfort, and information during the stressful and isolating first few months of the pandemic.

## Conclusions

We presented the findings of the MedConnect program, a medical student-initiated, phone interview-based follow-up program for COVID-19 patients discharged from the ED. Nearly one in five patients had another ED visit during the 14-day study period and more than half of those required hospitalization for worsening symptoms. There were statistically significant differences in multiple symptom trends and patient characteristics between those who returned to the ED, returned and were admitted, and did not return to the ED. Those who did not require hospitalization had a steady and rapid resolution of most of their symptoms, irrespective of age or comorbidities.
